# Epidemiology and Management of Hypertension and Diabetes Mellitus in Patients with Mild Autonomous Cortisol Secretion: A Review

**DOI:** 10.3390/biomedicines11123115

**Published:** 2023-11-22

**Authors:** Marta Araujo-Castro, Martin Reincke, Cristina Lamas

**Affiliations:** 1 Endocrinology & Nutrition Department, Hospital Universitario Ramón y Cajal, Colmenar Viejo Street km 9, 28034 Madrid, Spain; 2 Instituto de Investigación Biomédica Ramón y Cajal (IRYCIS), Colmenar Viejo Street km 9, 28034 Madrid, Spain; 3 Department of Medicine IV, University Hospital, LMU Munich, 80336 Munich, Germany; martin.reincke@med.uni-muenchen.de; 4 Endocrinology & Nutrition Department, Complejo Hospitalario Universitario de Albacete, 02006 Albacete, Spain; clamaso@sescam.jccm.es

**Keywords:** mild autonomous cortisol secretion, hypercortisolism, type 2 diabetes, hypertension, adrenalectomy

## Abstract

Mild autonomous cortisol secretion (MACS) is associated with a higher cardiometabolic risk than that observed in patients with nonfunctioning adrenal adenomas and in the general population. In patients with MACS, the excess of glucocorticoids affects various metabolic pathways, leading to different manifestations of metabolic syndrome and other comorbidities. Hypertension and diabetes mellitus are two of the most common cardiometabolic comorbidities associated with MACS, reaching a prevalence of up to 80% and up to 40%, respectively. In addition, they are the comorbidities that experienced a greater improvement after adrenalectomy in patients with MACS. Hypertension pathogenesis is multifactorial, including the coexistence of comorbidities such as obesity or diabetes and the role of the different polymorphisms of the glucocorticoid receptor gene, among others. Glucocorticoid-induced diabetes mellitus is mainly related to the detrimental effects of glucocorticoids on insulin-dependent glucose uptake in peripheral tissues, gluconeogenesis and insulin secretion. There are no specific recommendations for hypertension and diabetes treatment in patients with MACS. Thus, considering the similar underlying pathogenesis of hypertension and diabetes mellitus in overt and mild hypercortisolism, our recommendation is to follow this general stepwise approach: surgically remove the adrenal culprit lesion to induce remission from hypercortisolism; control hypercortisolism with steroidogenesis inhibitors; and treat elevated blood pressure or high glucose levels using carefully selected anti-hypertensives and glucose-lowering medications if blood pressure and glucose levels remain uncontrolled, respectively. In this review, we summarize the epidemiology, physiopathology and management of diabetes mellitus and hypertension in patients with MACS.

## 1. Introduction

Hypercortisolism can be considered a continuum both in terms of hormonal and cardiometabolic abnormalities [1]. Although a greater cardiometabolic risk has been described even in patients with non-functioning adrenal incidentalomas (NFAIs) than in the general population [2,3,4], the two main entities associated with endogenous hypercortisolism are mild autonomous cortisol secretion (MACS) and overt Cushing’s syndrome. Patients with adrenal incidentalomas who have MACS display a post-dexamethasone suppression test (DST) serum cortisol higher than 1.8 µg/dL without the specific clinical manifestations of overt Cushing’s syndrome, such as myopathy, bone fragility and skin fragility [5]. However, from a clinical point of view, it is important to note that both MACS and overt Cushing’s syndrome represent parts of an overlapping spectrum of hypercortisolism. Distinguishing clinically and biochemically a patient with an adrenal incidentaloma and MACS from a patient with overt adrenal Cushing’s syndrome is challenging. This was demonstrated in a recent study by Zhang et al. [6], in which a substantial percentage of patients with adrenal incidentalomas and MACS had central obesity (39.0% versus 83.3% in overt adrenal Cushing’s syndrome), supraclavicular and/or dorsocervical fat accumulation, (25.4% vs. 75.0%), typical skin changes (28.8% vs. 83.3%) and proximal muscle weakness (47.5% vs. 75.0%). Therefore, classifying a patient as having MACS requires a careful and critical assessment to not overlook overt Cushing’s syndrome.

The latest guidelines of the European Society of Endocrinology (ESE) and European Network for the Study of Adrenal Tumors (ENSAT) eliminates the differentiation between possible autonomous cortisol secretion (ACS) and ACS, considering that all patients with adrenal incidentalomas and a post-DST > 1.8 µg/dL should be classified as MACS [5] since a higher cardiometabolic risk and mortality was already observed above the threshold of 1.8 µg/dL in the DST, especially in women under 65 years of age [7]. For the management of MACS, the most important point is the evaluation of the cardiometabolic comorbidities potentially associated with hypercortisolism since the indication of surgery will be mainly dependent on the presence of these comorbidities and their grade of control [5]. Nevertheless, although these comorbidities usually improved after adrenalectomy in most of the cases [8], some patients had continued hypertension and/or diabetes mellitus after surgery. In addition, some patients were not candidates for surgery, with comorbidity management being the main challenge in these cases. 

Several mechanisms are responsible for the higher cardiometabolic risk in patients with hypercortisolism. Excess glucocorticoids affect various metabolic pathways, leading to the different manifestations of metabolic syndrome and other comorbidities [1]. Cardiovascular disease, hypertension and diabetes mellitus are the most prevalent comorbidities in patients with endogenous hypercortisolism. Additionally, diabetes mellitus and hypertension were found to be the cardiometabolic conditions that improved the most after adrenalectomy in MACS [8] and also after Cushing’s syndrome surgery [9].

We have performed a comprehensive review of MACS and its relationship with diabetes mellitus and hypertension using PubMed as the main database. Only articles published in English were included. In this review, we summarize the epidemiology and physiopathology of diabetes mellitus and hypertension in patients with MACS. We also offer some practical recommendations for the management of diabetes mellitus and hypertension in these patients.

## 2. Hypertension in Patients with MACS

### 2.1. Epidemiology

Patients affected with adrenal incidentalomas and MACS are at a higher risk of cardiovascular events and arterial hypertension than patients with NFAI and healthy controls [10,11,12,13,14,15,16,17]. Arterial hypertension is the most common clinical feature, being present in over 60% of MACS patients [10,11,12,13,14,15,16,17]. In this regard, a study by Patrova et al. [18], which included a total of 204 patients with NFAI and 166 with MACS, showed that patients with post-DST cortisol levels over 1.8 μg/dL were more frequently affected by arterial hypertension (39.2% in NFAI, 64.8% in possible ACS and 57.6% in ACS, *p* < 0.001) and required more anti-hypertensive drugs than those patients with NFAI. Another important study in this field is the ENSAT-EURINE study [19]. A total of 1305 patients with adrenal adenomas were prospectively included in this study; 50% had NFAI, 45% had ACS or possible ACS and 5% had overt Cushing’s syndrome. The authors found that patients with ACS and Cushing’s syndrome had a higher prevalence of arterial hypertension than patients with NFAI (adjusted prevalence ratios (aPR), 1.15 (CI 1.04–1.27) and 1.37 (1.16–1.62)) and required three or more antihypertensive drugs for blood pressure (BP) control more frequently than patients with NFAI (aPRs, 1.31 (CI, 1.02–1.68) for ACS and 2.22 (1.62–3.05) for Cushing’s syndrome) (Table 1). Supporting the results of these studies, a recent meta-analysis including 17,156 patients with adrenal incidentalomas found that patients with MACS had a higher prevalence of hypertension (RR = 1.24, 95% CI 1.16–1.32) than patients with NFAI [20]. Nevertheless, not all studies reported differences in hypertension prevalence among patients with NFAI, possible ACS and ACS [12,21,22].

Furthermore, it is noteworthy that arterial hypertension has been described as the comorbidity that improves the most after adrenalectomy in patients with adrenal incidentalomas and MACS. In this sense, Bancos´ meta-analysis [8], which includes a total of 584 patients with MACS and 457 with NFAI, found that surgically managed patients were eleven times more likely to have improved hypertension and four times more likely to have improved diabetes when compared to those managed conservatively.

### 2.2. Pathogenesis

The mechanisms involved in the pathogenesis of hypertension in patients with MACS are complex and still not fully clarified [23]. Nevertheless, the mechanisms seem to be quite similar to those observed in patients with overt Cushing’s syndrome, but at a lower degree [24]. The etiology of hypertension in cortisol excess is multifactorial and includes enhanced mineralocorticoid activity, changes in the renin angiotensin aldosterone system (RAAS), the vasoregulatory system, vascular remodeling, an increased synthesis of central neurotransmitters and an enhanced vascular response to catecholamines [25]. The role of locally activated RAAS in intraabdominal fat has been recently described [26]. One of the main mechanisms responsible for hypertension in overt Cushing’s syndrome is the saturation of the 11b-hydroxysteroid-dehydrogenase type 2 (11b-HSD2) enzyme due to high levels of cortisol, leading to cortisol binding to the mineralocorticoid receptor, causing sodium retention, volume expansion and hypertension [24]. In MACS, the degree of hypercortisolism is milder, so it should not affect the ability of 11b-HSD2 to convert cortisol into cortisone to a lesser degree. Related to this mechanism, it is noteworthy that even in patients with NFAI, post-DST cortisol levels were associated with a higher prevalence of hypertension and diabetes mellitus [27], suggesting that even patients with “NFAI” exhibit a hidden hypercortisolism [28].

The frequent coexistence of other comorbidities such as obesity, type 2 diabetes or metabolic syndrome may contribute to the development of hypertension in patients with MACS. Another point to consider is that the likelihood of developing hypertension is associated with the degree and duration of exposure to mild cortisol excess, as well as with tissue sensitivity to glucocorticoids. In relation to this last point, glucocorticoid sensitivity varies depending on the polymorphisms of the glucocorticoid receptor gene [29,30]. For example, the N363S polymorphism has been described to be more frequent in hypertensive patients, and it has been associated with hypertension in patients with suppressed cortisol after the 1 mg DST [29].

In addition, the coexistence of aldosterone and cortisol hypersecretion has been reported in about 30% of patients with primary aldosteronism (“Connshing syndrome”) [31,32]. Patients with cosecretion of aldosterone and cortisol have a higher prevalence of hypertension (odds ratio [OR] 7.7, 95% CI 2.64–22.32) than patients with MACS without primary aldosteronism. Thus, the mechanism leading to hypertension in patients with “Connshing syndrome” is multifactorial due to aldosterone and cortisol excess. In fact, a recent study found that plasma aldosterone plays a more important role in the pathogenesis of hypertension in patients with MACS than in those with overt Cushing’s syndrome, since they observed that plasma aldosterone concentration was significantly associated with elevated systolic BP among patients with MACS (adjusted difference (95% CI = 0.59 [0.19–0.99], *p* = 0.008) but not among those with overt hypercortisolism [33].

### 2.3. Management

There are no specific recommendations for hypertension treatment in patients with MACS, but there is a clinical guideline for hypertension management in patients with endogenous hypercortisolism [25]. Thus, considering the similar underlying pathogenesis of hypertension in overt and mild hypercortisolism, our recommendation is to follow this general stepwise approach: surgically remove the adrenal culprit lesion to induce remission from hypercortisolism; control hypercortisolism with steroidogenesis inhibitors; and treat elevated BP using carefully selected anti-hypertensives if BP remains uncontrolled.

The guidelines underscore the importance of controlling hypercortisolism through surgical or medical treatment because it leads to a reduction in morbidity, especially for hypertension and diabetes [20]. The surgical correction of MACS has also demonstrated a significant benefit for hypertension improvement [8]. There are two randomized clinical trials with results that were in favor of surgery [34,35]. In Toniato’s clinical trial [34], hypertension improved in 67% (12 of 18) after surgery, while it worsened in patients conservatively managed. In Morelli’s clinical trial [35], BP control improved in 68% of the operated patients vs. 13% of the patients in the conservative group (*p* = 0.001). When surgery is contraindicated, not possible or there are bilateral adrenal lesions, medical treatment for hypercortisolism control may be considered in those patients with associated comorbidities potentially linked to MACS [36,37]. However, data on the efficacy and safety of the medical treatment for MACS are still lacking since few studies have described their use in that setting [36]. In this way, it should be considered that since there are no specific recommendations for hypertension treatment in patients with MACS, the choice of the same medical treatment as for Cushing’s syndrome is considered an “off-label” treatment for MACS.

In Debono’s study [36], metyrapone normalized the abnormal circadian rhythm of cortisol when given in the late afternoon and evening (500 mg at 6 PM and 250 mg at 10 PM). Unfortunately, hypertension control was not assessed in this study. On the other hand, hypertension is one common secondary side effect of metyrapone, but at low doses (commonly used for MACS), the development or worsening of hypertension is not expected. In fact, in Debono’s study, only one patient out of the six MACS cases treated with metyrapone developed hypertension [36]. In summary, in this study, the use of timed evening doses of metyrapone reset the cortisol rhythm to normal. Thus, considering that the loss of the cortisol circadian rhythm may also be related to the pathogenesis of hypertension, especially with the non-dipping pattern, the correction of these alterations with metyrapone is expected to result in better BP control [23,24]. Ketoconazole, at a low dose (200–400 mg/day), also showed cortisol secretion normalization and BP amelioration in a patient with bilateral adrenal macronodular hyperplasia and mild Cushing’s syndrome [38]. Three different studies have evaluated mifepristone in the context of MACS; the drug led to an amelioration of insulin resistance, hypertension, quality of life and cardiometabolic parameters with good tolerability [39,40,41]. In a prospective open-label pilot study, six individuals with adrenal incidentalomas and MACS were treated with mifepristone 200 mg twice daily, and two of them had an improved mean 24 h BP from 143/75 to 135/67 mmHg and from 138/81 to 130/81 mmHg, respectively [39]. Similar results were obtained in the other two studies with mifepristone. Relacorilant was employed in seven patients with ACTH-independent Cushing’s syndrome, including patients with MACS, and in the low-dose group (100–200 mg/d; *n* = 17), 5/12 patients (41.7%) with hypertension achieved a good response. In the high-dose group (250–400 mg/d; *n* = 18), 7/11 patients (63.6%) with hypertension achieved a good response [42].

**Figure 1:**
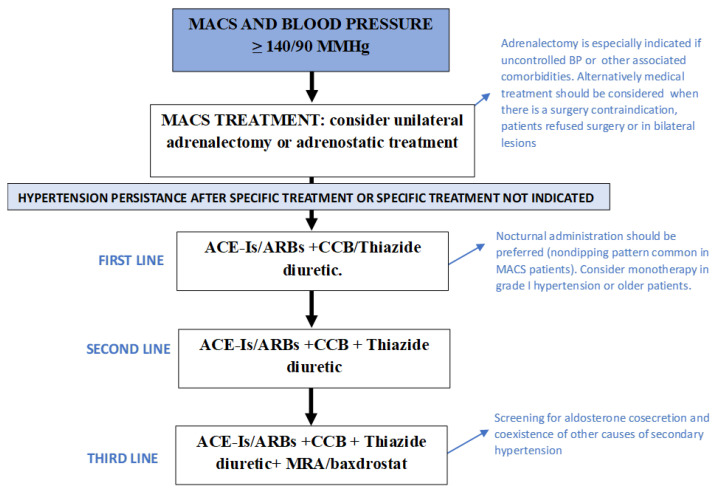
Recommendations for blood pressure control in patients with mild autonomous cortisol secretion. ACE-Is: angiotensin-converting enzyme inhibitors; ARBs: angiotensin receptor blockers; BP: blood pressure; CCB: calcium channel blockers; MACS: mild autonomous cortisol secretion; MRA: mineralocorticoid receptor blockers.

Due to the high cardiovascular risk linked to hypercortisolism, the goals of hypertension treatment should be to maintain BP levels below 130/80 mmHg. The adoption of a heart-healthy lifestyle is a fundamentally important approach to preventing or delaying the onset of hypertension, reducing elevated BP values, and reducing the associated increased cardiovascular risk [43]. As a generalized up-regulation of the RAAS is observed in patients with hypercortisolism, angiotensin-converting enzyme inhibitors (ACE-Is) or angiotensin receptor blockers (ARBs) should be considered the first-line approach to lowering BP levels in MACS patients [23]. This may be enough in the cases of grade I hypertension, high–normal BP, very high cardiovascular risk, frailty and older patients. However, the initiation of therapy with a two-drug combination is recommended for many hypertensive patients; thus, the combination with a calcium channel blocker (CCB), a beta-blocker or a thiazide diuretic should be considered [43]. If BP remains uncontrolled with two drugs at their maximum recommended dosage, a three-drug combination, usually a RAAS blocker + CCB + Thiazide/Thiazide-like diuretic, should be considered. Mineralocorticoid receptor antagonists (MRA) are usually used as a third-line treatment [23]. However, in patients with aldosterone cosecretion, MRA should be positioned as the treatment of choice for hypertension and cardiovascular risk management [44]. Baxdrostat, an aldosterone synthase inhibitor, is also a promising therapeutic option for patients with resistant hypertension and/or aldosterone oversecretion [45] (Figure 1).

## 3. Diabetes Mellitus in Patients with MACS

### 3.1. Prevalence

Hyperglycemia is a common associated comorbidity of MACS, being present in 15–40% of patients, depending on how MACS and hyperglycemia are defined [46] (Table 2). For example, in a study by Deutschbein T. et al. [7], the prevalence of type 2 diabetes (and other comorbidities) increased as a continuum from NFAI to possible autonomous cortisol secretion (ACS) and ACS patients (18.2%, 23% and 26.7%, respectively). These results are in line with those described by other authors [12,21]. Nevertheless, in the Patrova series [18], despite using the same definitions for NFAI, possible ACS and ACS, the prevalence of type 2 diabetes was higher in patients with possible ACS than those with ACS. However, in this study, patients with possible ACS were older than those with ACS. The risk of type 2 diabetes has been found to be proportional to the degree of hypercortisolism, the age of the patient and the size of the adrenal adenoma [46]. The association between DST levels and type 2 diabetes was even observed in NFAI patients [47].

In Elhassan’s meta-analysis [48], which included 4121 patients with MACS or with NFAI, the prevalence of type 2 diabetes was 18.1%, being twice as prevalent in patients with MACS (28.1%) than in patients with NFAI (14.4%). However, in Prete’s study [19], despite using the same post-DST threshold of 5.0 µg/dL for differentiating MACS and NFAI, the prevalence of type 2 diabetes reached 33.7% in patients with MACS. The prevalence of type 2 diabetes in this study increased in participants with overt Cushing’s syndrome (aPR), 1.62 (CI, 1.08 to 2.42), compared to NFAI, but not in patients with MACS. Nevertheless, in a subgroup analysis of people with type 2 diabetes, both patients with MACS and Cushing’s syndrome required insulin treatment more frequently than patients with NFAI (aPRs, 1.89 (CI, 1.01 to 3.52) and 3.06 (CI, 1.60 to 5.85), respectively). Similarly, in our Spanish cohort of 231 patients with MACS and 478 with NFAI, although we were not able to demonstrate differences in the prevalence of type 2 diabetes (27.7% vs. 22.6%, *p* = 0.137) between patients with MACS and those with NFAI, fasting plasma glucose values and glycated hemoglobin levels were significantly higher in patients with MACS [49]. Similarly, in a recent meta-analysis by Pelsma et al. [20], patients with MACS had a higher prevalence of hypertension (RR = 1.24, 95% CI 1.16–1.32), than patients with NFAI. Hyperglycemia in patients with MACS has been described as the cause of observed lipidic abnormalities. On the other hand, it has been described that in the presence of a subtle cortisol excess, there is no effect on lipid pattern in the absence of an altered glucose metabolism [50].

### 3.2. Pathogenesis

Chronic exposure to exogenous glucocorticoids is associated with an increased risk of developing type 2 diabetes mellitus, which is mainly due to the detrimental effects of glucocorticoids on insulin-dependent glucose uptake in peripheral tissues, gluconeogenesis and insulin secretion [46]. One of the major functions of cortisol is the regulation of glucose metabolism; it promotes gluconeogenesis through the activation of phosphoenolpyruvate carboxykinase and glucose-6-phosphatase in the liver [51].

**Figure 2:**
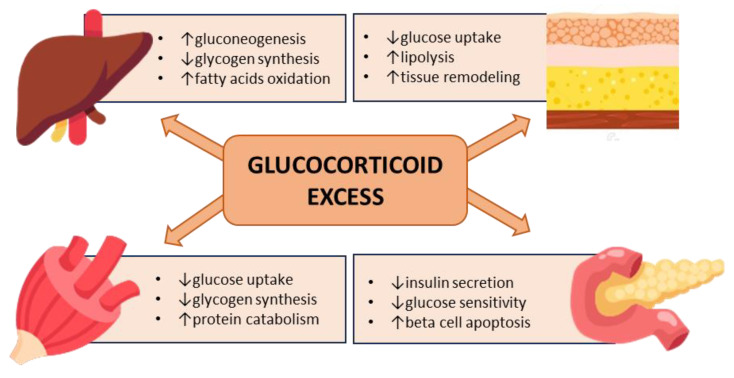
Hyperglycemia mediated by glucocorticoid excess.

In summary, chronic hypercortisolism may have pleiotropic effects on all major peripheral tissues governing glucose homeostasis, leading to insulin resistance and an impairment of insulin secretion, acting through genomic and nongenomic mechanisms in a context-specific and cell-/organ-dependent manner [46] (Figure 2). In addition to glucocorticoid excess, the impairment of circadian glucocorticoid secretion also contributes to metabolic abnormalities [52].

### 3.3. Management

There are several studies that demonstrate the benefit of surgical treatment in terms of improving the glucometabolic profile of patients with MACS [53,54,55]. In general, it is recommended to consider adrenalectomy in those patients with MACS, regardless of the grade, who present associated comorbidities such as type 2 diabetes, hypertension or osteoporosis, especially if the patient is young and the comorbidities are not adequately controlled [5].

In addition to several observational studies that demonstrate the benefit of adrenalectomy [53,54,55], two randomized clinical trials have been carried out, and their results were also in favor of surgery [34,35]. In Toniato’s clinical trial [34], 23 cases were included in the surgery group and 22 patients in the conservative management group, describing an improvement in type 2 diabetes in 62.5% (5 of 8) after surgery, while diabetes worsened in patients who were conservatively managed. Morelli’s clinical trial [35] included 25 patients who underwent adrenalectomy and 30 who were conservatively managed. An improvement in glucometabolic control was reported in 28% of the adrenalectomy group vs. 3.3% in the conservative group (*p* = 0.02).

In those patients with contraindications, bilateral lesions or who reject surgery, steroidogenesis inhibitors could be used [37]. The greatest benefit in terms of the glycemic profile has been reported for mifepristone, an oral non-selective GR antagonist, which is not available in the EU. Some of these studies reported improvements in hypertension and glucose tolerance in 38% and 60% of patients with overt Cushing’s syndrome, respectively [56]. There is also evidence of the benefits of mifepristone use in patients with MACS. In a prospective open-label pilot study including six patients with MACS, a dose of 200 mg twice daily led to a reduction in the insulin AUC > 7237 pmol/L.min in five out of six individuals, and in two patients, this showed a clinically significant cardiovascular benefit [39]. Another study, including eight patients with either unilateral or bilateral adrenal nodules with MACS treated with a dose of 300 mg per day, also observed that there was a significant reduction in fasting glucose levels and insulin resistance, as measured by HOMA-IR, in six of the eight patients enrolled [40]. A third study included a retrospective series of four patients with bilateral macronodular adrenal hyperplasia and MACS; again, mifepristone led to an improvement in glycemic and hypertension control in all the patients [41].

Another tested treatment in patients with MACS is relacorilant, a selective glucocorticoid receptor modulator. In an open-label, phase 2 dose-finding study, seven patients with ACTH-independent Cushing’s syndrome, including patients with MACS (based on the definition of DST > 5 µg/dL and low or suppressed ACTH), were included. In terms of the main findings, in relation to diabetes control, they reported that in the low-dose group (100–200 mg/d; *n* = 17), 2/13 patients (15.4%) with hyperglycemia had a good response. In the high-dose group (250–400 mg/d; *n* = 18), 6/12 patients (50%) with hyperglycemia had a good response [42].

Similarly, a subanalysis of the SONICS clinical trial comparing diabetic and non-diabetic patients with overt Cushing’s syndrome [57] found that a treatment with levoketoconazole led to a sustained normalization of urinary free cortisol (46% vs. 33%, *p* = 0.02) and an improvement in glycemic control (the mean HbA1c decreased from 6.9% at baseline to 6.2% in patients with type 2 diabetes and from 5.5% to 5.3% in patients without type 2 diabetes) that was more pronounced in patients with type 2 diabetes. Nevertheless, this drug was not used in patients with MACS.

Regarding cortisol-lowering medication for the treatment of MACS with the aim of improving diabetes control, as occurs for hypertension management in MACS, it should be considered that these medications can only be prescribed as “off-label” treatments for MACS.

If specific medical or surgical treatment of MACS is not considered or possible, the management of diabetes mellitus should be based on the general recommendations for the management of type 2 diabetes [58]. However, a special priority should be given to therapeutic options with a protective cardiovascular profile that act by reducing insulin resistance. In this regard, metformin continues to be the first-line therapy due to its efficacy and insulin sensitizer mechanism of action [59].

## 4. Conclusions

Hypertension and diabetes mellitus are two of the most common cardiometabolic comorbidities associated with MACS, reaching a prevalence of up to 80% and up to 40%, respectively. In addition, they are the cardiometabolic conditions that improve the most after adrenalectomy in patients with MACS.

## Figures and Tables

**Table 1:** Prevalence of hypertension in patients with mild autonomous cortisol secretion (ACS and possible ACS) compared to patients with nonfunctioning adrenal incidentalomas.

Study	NFAI	Possible ACS	ACS	*p* Value
Prete A, 2022 [19]	416 (64.1%)	339 (75.2%)	107 (76.4%)	<0.05
Deutschbein T, 2022 [7]	1186 (58.6%)	944 (74%)	179 (75.2%)	NR
Patrova J, 2017 [18]	80 (39.2%)	83 (64.8%)	19 (57.6%)	<0.001
Dalmazi Di, 2014 [21]	73 (57%)	36 (61%)	9 (90%)	0.18
Dalmazi Di, 2012 [12]	149 (73.4%)	82 to 78% *	18 (94.7%)	0.173

ACS: autonomous cortisol secretion (cortisol post-DST > 5 µg/dL); possible ACS (cortisol post-DST between 1.8 and 5 µg/dL); NFAI: nonfunctioning adrenal incidentalomas (cortisol post-DST ≤ 1.8 µg/dL); NR: not reported. * 82% for those patients with high urinary free cortisol (UFC) or basal plasma ACTH < 10 pg/mL and 78% with normal UFC and basal plasma ACTH.

**Table 2:** Prevalence of diabetes mellitus in patients with mild autonomous cortisol secretion compared to patients with nonfunctioning adrenal incidentalomas.

Study	NFAI	Possible ACS	ACS	*p* Value
Prete A, 2022 [19]	171 (26.4)	145 (32.2)	47 (33.7)	>0.05
Deutschbein T, 2022 [7]	365 (18.2%)	288 (23%)	62 (26.7%)	NR
Patrova J, 2017 [18]	25 (12.3%)	26 (20.3%)	3 (9.1%)	0.111
Dalmazi D, 2014 [21]	18 (14%)	17(29%)	4 (40%)	0.02
Dalmazi D, 2012 [12]	31 (15.2%)	18 to 37% *	8 (42.1%)	0.004

ACS: autonomous cortisol secretion (cortisol post-DST > 5 µg/dL); possible ACS (cortisol post-DST between 1.8 and 5 µg/dL); NFAI: nonfunctioning adrenal incidentalomas (cortisol post-DST ≤ 1.8 µg/dL). * 37% for those patients with high urinary free cortisol (UFC) or basal plasma ACTH < 10 pg/mL and 18% with normal UFC and basal plasma ACTH.

## Data Availability

Not applicable.
